# Chemical analysis of extracellular vesicles by synchrotron-based X-ray imaging and scattering techniques: a review and perspective

**DOI:** 10.3389/fbioe.2025.1769106

**Published:** 2026-01-12

**Authors:** Li Huang, Yan Chen, Xiutian Guo, Limin Zhou

**Affiliations:** 1 Department of Anorectal Surgery, Shanghai Municipal Hospital of Traditional Chinese Medicine, Shanghai University of Traditional Chinese Medicine, Shanghai, China; 2 Shanghai Synchrotron Radiation Facility, Shanghai Advanced Research Institute, Chinese Academy of Sciences, Shanghai, China

**Keywords:** chemical analysis, extracellular vesicles, small angle X-ray scattering, soft x-ray microscopy, X-ray photoelectron spectroscopy

## Abstract

Extracellular vesicles (EVs) are nanoscale lipid bilayer-enclosed particles released by cells, which have been explored as pivotal mediators for intercellular communication, biomarkers for diseases and nano-carriers for drug delivery. Unraveling their structural and chemical heterogeneity is crucial for understanding the biogenesis, cargo sorting, and functional mechanisms of EVs. However, by far it remains challenging to characterize the intrinsic physicochemical properties of EVs due to their varied intracellular origins, poly-disperse size distribution and dynamic membrane organization. Conventional imaging and light scattering methods either lack the chemical sensitivity or suffer from labeling artifacts. Here in this review, we summarize research work using synchrotron-based X-ray imaging and scattering techniques to resolve the chemical structural complexity of EVs with intrinsic chemical specificity and enhanced sensitivity. The feasibility and effectiveness of X-ray imaging and scattering tools on quantifying critical structural parameters of EVs including morphology, core-shell and bilayer structure is discussed. We hope it will inspire future in-depth work to bridge the gap between structural and biological functionality in EVs research.

## Introduction

Understanding the fundamental physicochemical properties of biomaterials including their morphology, composition and stability in biological fluids, is the prerequisite to explore their therapeutic effects and potentials in targeted drug delivery. Extracellular Vesicles, as the cell released heterogeneous lipid-based nanoparticles with size ranges from 30 nm to 1000nm, have been found to serve as fundamental biological mediators and intercellular communicators through the transfer of proteins, nucleic acids, and lipids (Kalluri and LeBleu, 2020; Wang et al., 2023). In recent years, EVs are actively explored as promising natural nano-sized carriers (Feng et al., 2023), versatile biomaterials (Carney et al., 2025) and biomarkers (Chen et al., 2024; Hu et al., 2025) for therapeutic and diagnostic approaches. Their structural properties like size, topology, membrane fluidity, molecular composition and mechanical stiffness directly govern functional outcomes in immunity, cancer metastasis, and tissue regeneration (Manno et al., 2024).

Consistent research efforts have been devoted to uncover their unique physicochemical landscape and establish the connections between structure and biological functions. Due to the small sizes of EVs, high resolution imaging techniques is among the most important methods to reveal the spatial-temporal property of EVs. Electron microscopy including Scan Electron microscopy (SEM) and Transmission Electron microscopy (TEM) has become the common tools to acquire the basic size and morphology of EVs both *in vitro* and *in vivo* (Chuo et al., 2018; Verweij et al., 2021). Atomic force microscopy (AFM) is very suitable to imaging biomaterials in liquid environment and has been applied to illustrate the mechanical properties of EVs as well as the heterogeneous protein assembly within membrane structure (Hardij et al., 2013; Wang et al., 2020). High speed AFM revealed the“Y-like” conformation of exosome markers IgG co-localized with small EVs with diameter below 100 nm instead of the larger ones (Sandira et al., 2025). Optical microscopy with high resolution is another common method used to track the merging and release events of EVs and their interactions with cells, proteins and others, although fluorescence-based labeling is always necessary (He et al., 2023; Hu et al., 2025). The light-scattering techniques like dynamic light scattering (DLS) and nanoparticle tracking analysis (NTA) (Gardiner et al., 2013; Stetefeld et al., 2016) has been commonly used to determine the size distribution and number density of biomolecules including EVs in aqueous solutions.

**FIGURE 1:**
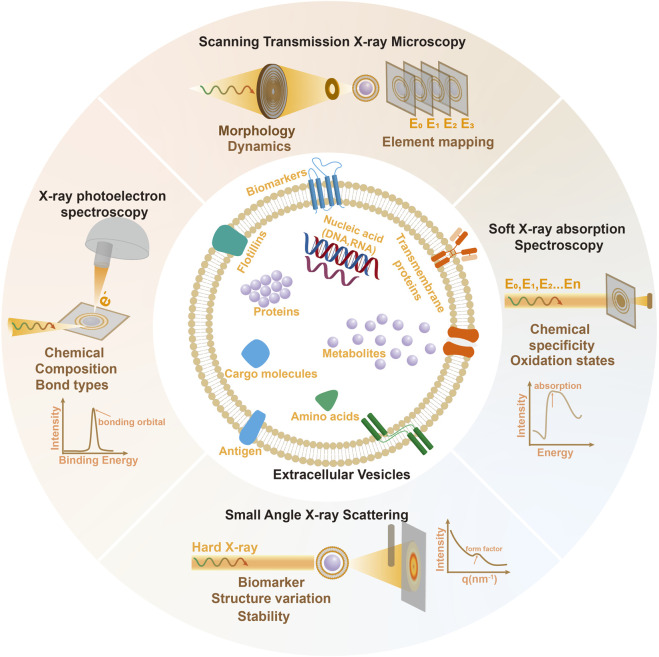
Schematic of the principle of Synchrotron based X-ray imaging and scattering methods and basic structure and physicochemical properties of EVs probed by these techniques.

Despite these advances, precise characterization of the fundamental properties like topology heterogeneity, membrane asymmetry and structure of bio-molecular corona of EVs remains challenging. Part of the reasons comes from the limitations of conventional methods for EVs study such as lack of chemical sensitivity and the need of pre-labeling or fixation which will hinder the observation of EVs-related activities under native biophysical states. Synchrotron-based X-ray imaging and scattering techniques is emerging fast in the past decades with the establishment of over 35 major synchrotron light-source facilities worldwide. Advancements in both X-ray instruments and techniques significantly enhanced the ability to study biomaterial surfaces, cellular components, and subcellular structures with high accuracy and minimal disruption. In this work, we have reviewed x-ray imaging and scattering methods that are suitable for EVs research, as illustrated in Figure 1, their basic principle, current advances and future potentials in revealing vital physico-chemical properties of EVs are presented.

## Challenging and limitations of conventional EVs analysis methods

Since the interactions between EVs and the cellular microenvironment like cargo transport, targeted delivery or crossing biological barriers is closely connected to some specific molecules or proteins attached on the EV membrane, the capability of identifying and chemical analysis of these bioactive molecules is a priority task. However, for conventional imaging and scattering techniques such task is quite troublesome. Due the low signal-to-noise ratio of biological specimens under electron irradiation, EV imaging by SEM/TEM requires careful sample preparation, including dehydration, fixation, and metal staining which can distort EV morphology and obscure native composition. Since low temperature (liquid nitrogen ∼90K) can better preserve the quality and morphology of biological sample, cryo-EM is increasingly adopted to study EVs under native hydrated state (Yuana et al., 2013; Morandi et al., 2022). AFM provide high-resolution topographic profiles and biomechanical information, yet it often lacks chemical specificity. Conventional fluorescence microscopy either lacks the chemical sensitivity or suffers from labeling artifacts.

Light-scattering methods like DLS and NTA is generally convenient, no-invasive and label-free. They can capture the size distribution and concentration but are limited to differentiate between EVs and other particles, such as protein aggregates or macromolecules. Recent study suggest that different NTA devices may have advantage and disadvantage in the accurate estimation of the EV size and concentration, and both failed to yield realizable data on the smaller EVs with diameter below 60 nm (Bachurski et al., 2019). One worthwhile choice is combining high-resolution microscopy like TEM with the scattering methods to avoid potential errors and acquire reproducible measurements of EVs. Meanwhile the heterogeneity of EV populations often leads to the overinterpretation of data and inconsistent claims which highlight the necessity for single EV research (Su et al., 2025). In general, the current research methods all struggle with analyzing heterogeneous EV populations and lacks the ability to provide detailed compositional or structural information. These limitations underscore the need for more advanced, label-free, and chemically specific techniques, such as synchrotron-based X-ray imaging and scattering, which offer a more accurate and comprehensive understanding of EV physico-chemistry in native conditions.

### Soft X-ray spectro-microscopy study of extracellular vesicles

Soft X-rays interact strongly with light elements via X-ray absorption and fluorescence, making them ideal for studying biological specimens (e.g., cells, proteins, lipids) without extra labeling. The soft x-rays absorption spectrum can provide spectroscopic information about the specimen. When choosing incident photon energy at specific X-rays absorption edge like K edge of Carbon (∼284eV), Nitrogen (∼400eV), Oxygen (∼540eV) or L3 edge of Calcium (∼364eV), Iron (∼708eV), Copper (∼931eV), Zn (∼1020eV), the strong absorptions around these edges are transformed to the spatial distribution and chemical states of the selected element compounds. This is particularly useful to identify specific functional groups or biomarkers within biological materials.

For direct imaging, synchrotron-based Scanning Transmission X-ray Microscopy (STXM) is a powerful nanoscale probe with chemical sensitivity which is well-suited for the characterization of nanomaterials. This soft X-ray based imaging technique has become the routine methods among worldwide synchrotron radiation facilities (Hitchcock, 2015; Feggeler et al., 2023; Shin et al., 2018; Wu et al., 2025) and made available to a broad range of scientific fields. Nanometer resolution (∼30 nm) is obtained by focusing the soft x-rays into spot size well below100 nm via Fresnel zone plate lenses as illustrated in Figure 1.

Another important techniques is the newly developed soft X-ray spectro-ptychography (Hitchcock, 2015) which combines soft X-ray spectroscopy with ptychography to enable chemically specific analysis of nanomaterials. Compare to traditional STXM, the ptychography approach is performed by scanning the sample with focused beam to acquire overlapping diffraction patterns and then using iterative phase retrieval algorithms to reconstruct into real space images and phase maps. The pectro-ptychography has higher spatial resolution (sub-10 nm), lower radiation dose and better image quality (Hitchcock et al., 2024). By combining STXM and ptychography at a specific X-ray absorption edge, one can produce a 3D dataset combining spatial and spectral data, enabling the chemical mapping of the probed sample area. Significant advances of soft x-ray microscopy studies on biological materials have been summarized in several reviews (Hitchcock et al., 2005; Hémonnot and Köster, 2017; Cao et al., 2022).

To direct visualize biological specimens like bacteria or single cell by soft x-ray microscopy, extreme care should be taken. One of the main reason comes from the radiation damage which is capable of breaking down C=O bonds upon reaching critical radiation dose ∼1.5*10^7^ Gy (Beetz and Jacobsen, 2003); another reason is the low penetration depth of soft X-rays in organic compounds (usually ∼1um), which requires very thin specimens. In recent years, progress like the implementation of fast fly-scan mode of STXM (Sun et al., 2021) and design of liquid-enclosing fluidic cells which enables living cells imaging (Yu et al., 2018) are made to overcome these difficulties.

**FIGURE 2:**
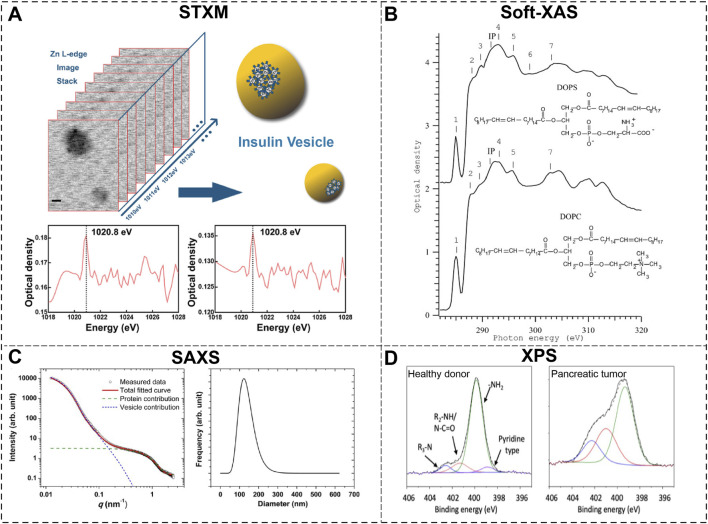
Characterization of extracellular vesicles by Synchrotron-based X-ray Techniques. **(A)** Scan Transmission X-ray Microscopy (STXM) study of insulin vesicle unveils the distribution of Zn^2+^ around the vesicle structure (Guo et al., 2022); **(B)** Soft x-ray absorption spectroscopy (XAS) of two types of lipids at carbon K edge (Nováková et al., 2008); **(C)** Small angle X-ray scattering (SAXS) results provide traceable size distribution of an erythrocyte-derived EV sample (Varga et al., 2014); **(D)** X-ray Photoelectron Spectroscopy (XPS) study revealed significant differences in the nitrogen environment of EVs isolated from pancreatic cancer patients and healthy donors (Sancho-Albero et al., 2023).

Application of soft X-ray spectro-microscopy in the study of EVs may open a whole new window for non-invasive nanoscale mapping of biochemical distributions inside or surrounding EVs. In Figure 2A, the precise content of Zn^2+^ and the location of insulin within single extracellular insulin vesicles are direct visualized by STXM and ptychography phase images at Zn L edge (∼1020eV) (Guo et al., 2022). Furthermore, the three dimensional (3D) structural mapping of spatial distribution of insulin vesicles inside pancreatic beta cells was constructed by combing X-ray ptychography and equally sloped tomography (EST) algorithm. The chemical state of lipid and proteins within the EV membrane is vital to understand its functionality and interactions. Figure 2B presents the soft x-ray absorption spectra of spectra of lipid bilayers of two common types of unsaturated phospholipids (Nováková et al., 2008). Features in the absorption spectra can be clearly attributed to specific bonds or resonances from functional groups like carboxylate and carbonyl groups. These results demonstrate the capability of soft X-ray spectra-microscopy in quantitative analysis of the chemical states of elements in heterogeneous EV structure.

Generally, soft X-ray spectro-microscopy could provide element-specific info about the internal structure and composition of EV membrane, as well as its stability and dynamic evolution at different biochemical conditions like PH, temperature and concentration of salt solution. The presence of nucleic acids (RNA/DNA), metabolites, or enzymes inside EVs may also be visualized under their native state. The biomolecular corona of EVs is the dynamic layer of biomolecules such as proteins, lipids, carbohydrates, and nucleic acids which adsorb onto the surface of EVs (Kalluri and LeBleu, 2020; Manno et al., 2024). Another potential research direction of EVs via soft x-rays may be its capability to obtain the chemical map of the biomolecular corona surrounding EVs, which is essential to understand how EVs interact with their biological environment.

By further developing *in situ* soft X-ray spectroscopic techniques, one may capture the process when proteins and other molecules from biological fluids adsorb onto EV surfaces in physiological environments. However, challenges like exposure control to minimize radiation damage and preparation of thin sample still exists. Meanwhile, reconstructing large datasets of diffraction patterns of EVs for real-time data analysis is yet to be developed. In the near future, high performance computing and machine learning inspired algorithms will offer unprecedented opportunities to accelerate reconstruction process and improve data quality.

### Small angle X-ray scattering study of extracellular vesicles

SAXS have been widely used to characterize the structural properties of diverse systems range from biological macromolecules to nanoparticles in liquid suspensions with 1–2 nm resolution (Li et al., 2016; Da Vela and Svergun, 2020; Tants and Schlundt, 2023; Caselli et al., 2024). As in Figure 1, small Angle X-ray Scattering (SAXS) collects the intensity of the scattered X-rays at very small angle ( θ=2∼5° ) and a fixed position using X-ray area detector. The scattering profiles have a reciprocal relation to the real space length, depending on the size, shape, and arrangement of structures at the nanoscale (typically 1–1,000 nm).

The momentum transfer between the incident light (wave-vector $\vec{k_{0}}$ ) and elastically scattered radiation at an angle 2 θ (wave-vector $\vec{k}$ ) are defined as scattering vector $q=\vec{k}-\vec{k_{0}}$ , and the modulus of the scattering vector can be expressed by the scattering angle θ and wavelength as $\left|q\right|=q=4\pi \sin\theta /\lambda$ . In real space, the modulus is further related to inversed length scale of the scattering object by q∼2π/D . To acquire the one-dimensional scattering profile, the radiation intensity within the image collected by the area detector is spherically averaged according the sample detector distance (SDD) and pixel size. For macromolecules in the solution, the total scattering intensity in the dilute solution is the linear combination of all the macromolecules and solvent molecules. Therefore, background subtraction is always necessary and the scattering curve from only macromolecules I(q) is obtained by subtracting the scattering curve of buffer without macromolecules by $I\left(q\right)=I_{sol}-I_{buffer}$ .

To determine the size and morphology of the macromolecules or nanoparticles, the Guinier law and the Porod’s law are typically applied for SAXS analysis. The Guinier law suggests that the scattering intensity is simplified at the small q region as $I\left(q\right)=I\left(0\right)e^{-{R_{g}}^{2}q^{2}/3}$ ,with $I\left(0\right)$ the intensity from zero scattering angle which represent the excess electron in the macromolecule as compared to the buffer. This approximation provides a straightforward yet accurate way to determine the size of one particle. The radius of gyration $R_{g}$ can be viewed as the effective size of macromolecule, for example, in monodisperse solid spheres, $R_{g}=\sqrt{3/5}R$ with the sphere radius R. The Porod’s law reflect the asymptotic behavior at the high q range ( $q\gg \pi /R_{g}$ ), in which the scattering solely arises from interfacial boundaries. The scattering intensity follows a power law of $q^{-1},q^{-2},q^{-4}$ for 1D, 2D and 3D objects with sharp electron density contrasts. Overall, the Guinier approximation at low q range probe the overall size/shape, while the power law scattering form Porod approximation at high q characterize interfaces between well-defined phases at nanoscale. However, it is noted that for more flexible and disordered macromolecules, the above empirical models could only extract asymptotic information from the SAXS data. Model analysis with *ab initio* methods (Mertens and Svergun, 2010; Petoukhov et al., 2012), machine learning methods (Franke et al., 2018; Röding et al., 2022), ensemble optimization method (Mertens and Svergun, 2010; Tria et al., 2015)and correlation function approach (Franke et al., 2015) and so on have been developed and continuously optimized for in-depth structural characterization of biological macromolecules in solution.

SXAS is viewed as a promising and state-of-the-art technique in the research of biological macromolecules and their interactions (Da Vela and Svergun, 2020; Chen et al., 2023). It has been applied to characterize the both the size and morphology of vesicle-like structures in the early 1990s. The effects of proteins presence and the curvature of membrane on the scattering profile of lipid vesicle were studied (Bouwstra et al., 1993) and it is revealed that membrane curvature or the variation in membrane thickness could reduce the first node in the scattering curve. The size and structure of purified synaptic vesicles were characterized which refined the structural information on their protein layer and lipid bilayer at the supramolecular level (Castorph et al., 2010). In Figure 2C, Varga et al. (2014) further unraveled the traceability of SAXS in the size determination and structure recognition of EVs when compared with techniques like electron microscopy and nanoparticle tracking analysis. The “core-shell” model is further applied to fit the scattering curve of EVs: the shell represents the phospholipid bilayer and the core represent the inner space of vesicles containing proteins and nucleic acids. Their fitting results match well with the SAXS data with two features of vesicle contribution at lower q and free proteins contribution at higher q (0.5–1.5nm^-1^). These results clearly reflect the distinct membrane-encapsulated structure of vesicles and established a pioneer theoretical model of EVs.

Apart from size and morphology determination with SAXS, in-depth studies on the physicochemical properties of EVs are still insufficient and valuable information might be extracted. For example, the Kratky plot of ( $q^{2}I\left(q\right)\;vs.q$ ) or its dimensionless form which resolve the conformational order in biomolecules, can be utilized to reveal the folded/unfolded lipid structure, order/disordered protein cargo within the vesicles as well as the flexibility of EV membrane. Since SAXS is very sensitive to conformational changes and electron contrast variation with sub-nm resolution, it opens up a unique window to study EV involved events like aggregation, fusion and cargo release. Analysis of the variation at high q range would also provide information on the thickness of hydration shell and the density of protein corona. Furthermore, EVs labeling is an important process to track the biological processes and functions of EVs in cells and *in vivo*, yet currently it is still challenging to evaluate the labeling efficiency and reliability (Hu et al., 2025). SAXS has proven to be a powerful tool to study the interactions between nanoparticles or surfactant-macromolecules (Chen et al., 2023). Therefore, when EVs surface are labeled by nanoparticles, quantum dots (QDs) or certain antibody, it’s very likely that the structure change of EV membrane and label probes can be unveiled by the contrast variation analysis of SAXS.

### X-ray photoelectron spectroscopy study of extracellular vesicles

X-ray Photoelectron Spectroscopy (XPS) is a photon in/electron out analytical technique which is surface sensitive (1–10 nm) and can provide quantitative element-specific information of materials. By measuring the kinetic energy of electrons $E_{k}$ photo-emitted from the sample irradiated with mono-energetic soft X-rays, the electron binding energy $E_{B}$ referenced to the Fermi level of the sample can be deprive by: $E_{B}=h\nu -E_{k}-\phi$ ,where hν is the X-ray photon energy, ϕ is the spectrometer work function which is normally a constant. The electron binding energy is referred to the energy required to remove an electron from a specific atomic orbital within the element (e.g., the C 1s, O 1s, and N 1s orbitals). Therefore, XPS is capable of providing unique fingerprint information about the chemical environments of elements within the sample, including the different bond types (e.g., C-C, C=O, C-N), oxidation states and also the chemical shifts arise from surface binding or intermolecular binding.

Due to its surface sensitivity with nanometer penetration depth, XPS is capable of measuring only the EV membrane without interference from the biomolecules inside EV. This is particularly useful to characterize the interfacial properties of engineered EVs or nanoparticles attached to EV membranes. María et al. reported the XPS study on the nitrogen environment of EV membranes from cancer and healthy cells (Sancho-Albero et al., 2023). The N chemical environment is viewed as an indicator of the relative abundance of pyridine-type bonding, primary, secondary and tertiary amines. As in Figure 2D, comparison of the relative abundances in XPS results between healthy donor and pancreatic tumor shown significant increase in the region concerning secondary amines (R2NH/N-C=O), indicating significant changes in the lipidomic profile of their EV membranes. Pan et al. utilized XPS to characterize both the cleavable lipid probes PO_4_^3—^spacer DNA-cholesterol and Zr-based Metal-organic frameworks which are constructed for rapid and effective EV enrichment and isolation from plasma fluid (Pan et al., 2022). These works demonstrated the potentials of XPS being a fast and no-invasive technique to characterize the chemical composition of lipid and proteins in EV membrane.

## Conclusion and perspective

The physicochemical properties of EVs including size, composition, interfacial chemistry, membrane topology and mechanic stiffness, have substantial influence on the outcome of intercellular communication, target delivery and signal transduction where EVs interact with the extracellular environment. The extreme small size and heterogeneous origins of EVs poses challenges to accurate describe their membrane structure, cargo distribution and surface chemistry. As compared in Table 1, conventional techniques such as EM, AFM, NTA and fluorescence imaging have long been relied upon for EV characterization, yet they each present significant limitation from lack of chemical specificity, labeling artifacts to overlook of delicate structure. Therefore, to fully capture such complexity of EVs under hydrated and native environments, there is an urgent need for advanced “non-invasive” techniques that can probe EVs at the nanoscale and acquire detailed chemical and morphological information while maintaining structural integrity.

**TABLE 1:** Comparison of chemical analysis capability of EVs among different imaging and spectroscopy techniques.

Technique	Spatial resolution	Temporal resolution	Chemical sensitivity	Label-free	Post-data analysis	Sample requirement
Fluorescence microscopy	250 m	ms ∼ s	Specific molecules	No	Image process	Live, fixed
Electron microscopy (SEM/TEM)	∼1 nm	∼min	Elements	Hardly	Image process	Cryo, fixed
Scan transmission X-ray Microscopy(STXM)	∼30 nm	∼min	Bonds chemical states	YES	Image reconstruction	Cryo, fixed
Soft X-ray absorption spectroscopy(XAS)	∼100 um	∼min	Bonds chemical states	YES	Spectral analysis	Cryo, fixed
X-ray photoelectron Spectroscopy(XPS)	∼100 um	∼min	Bonds chemical states	Hardly	Spectral analysis	Fixed
Small angle X-ray scattering(SAXS)	∼1 nm	∼s	Low	YES	Image reduction, modeling	Solution
Nanoparticle tracking analysis (NTA)	∼20 nm	∼s	Low	YES	Scattering data modeling	Solution
Atomic force microscopy (AFM)	∼1 nm	∼min	Low	YES	Image process	Solution

The emergence of synchrotron-based X-ray imaging and scattering techniques may fill the gap in EVs characterization and serve as the powerful alternative to conventional methods. Soft X-ray spectro-microscopy including XAS, STXM and ptychography could exploit intrinsic contrast of carbon, nitrogen, and oxygen 1s→π* transitions, thereby resolving membrane composition, protein/lipid ratio, and oxidative states of phospholipid headgroups with single EV particle chemical mapping at a spatial resolution down to ∼20 nm. Complementarily, small-angle X-ray scattering (SAXS) yields unbiased size distributions, membrane curvature profiles and structural variation under different biological conditions with the time resolution of minutes or even seconds. The surface sensitive XPS is also ideal to analysis the oxidation states of lipids and proteins within EV membrane as well their interaction with biomarkers or other nanoparticles.

The chemical analysis capability of X-ray imaging and scattering techniques may also be helpful to elucidate the therapeutic and diagnostic potentials of EVs for various diseases like cancer and immune-mediated inflammatory diseases. For instance, direct imaging of engineered EVs with antibodies via soft x-ray ptychography could reveal the binding efficiency of antibodies on EV membrane and their targeting effects to cancer or immune cells. The morphology analysis by SAXS will enable real-time tracking of how EVs interact with biomacromolecules like antibodies, nanoparticles and transmembrane proteins in the EVs-based drug delivery systems. The chemical sensitivity of XPS may also aid the efforts of identifying the specific lipids or biomarkers on the EV membrane that are different in healthy and cancer cell-derived nanovesicles, thus severing as potential diagnosis tools.

Despite these advantages, challenges such as radiation damage, sample preparation, and data processing remain to be addressed. For example, beam-induced radiation damage and water-window absorption necessitate careful optimization on x-ray dwell-time and low-dose protocols; sample heterogeneity mandates microfluidic isolation strategies to mitigate aggregation artefacts; and data volumes from phase retrieve of soft X-ray spectro-ptychography and *in situ* SAXS demand reproducible denoise reconstructions and machine-learning algorithms. Nevertheless, with the synchrotron facilities continue to evolve, improvements in beam coherence, brightness, and energy resolution will enable even higher spatial and chemical resolution, allowing researchers to probe EVs at the sub-nanometer scale with greater precision. The integration of advanced detectors and real-time data processing will significantly enhance the ability to analyze dynamic processes such as EV secretion, trafficking, and interactions with target cells in physiological environments. Future development may also enable multi-modal imaging, combining X-ray techniques with other spectroscopic or imaging methods like AFM/TEM and Raman to provide new insights on EVs composition and their cellular behaviors, paving ways to advance our understanding of EV biology and enable precise, data-driven applications in medicine and bioengineering.
